# The Causes and Treatment of Convulsive Affections of Children

**Published:** 1840

**Authors:** Alfred Millikin

**Affiliations:** Courtland, Alabama


Art IX.—*On the Causes and Treatment of Convulsive Affections of Children.* By Alfred Millikin, M. D., of Courtland, Alabama.

The remote causes productive of convulsive phenomena in children, are of a multiplex character. They may proceed from cold, producing local inflammation of some organ or tissue, the brain becoming affected secondarily; in which case there is a preternatural afflux of blood to that organ; its vessels being in a debilitated condition, do not contract and circulate freely the blood they receive, plethora, irritation and inflammation being the direct consequence, which is succeeded by a train of diseased action naturally growing out of this morbid condition of the parts, of which coma, convulsions, &c. are among the most common. In children, one form or the other is almost an invariable sequence to this condition of the brain. In other cases, the brain is the first organ to complain, being, perhaps, relatively, the weaker organ. This cause, productive of so much disease and death, does not exercise its deleterious influence upon individuals of any particular districts of country; but its action on man, in the different stages of life, is greatly diversified. In the adult, it is more likely to produce inflammation of the serous and mucous membranes, lining the close and open cavities, and not less frequently the capsular ligaments of the joints, the tendons, the muscular substance, &c. In children, the brain seems to be the organ upon which its morbid influence is mainly expended, either directly or indirectly. Whether this be attributable to the size of the organ, relatively large, at that period of life, or to its greater excitability, it is not easy to determine, but the fact is one well known to the profession.

*Repelled cutaneous eruptions* are also sources of this alarming disease—as of discharge from different parts of the scalp, or the sudden disappearance from the skin of any of the exanthemata.

*Intestinal worms* are also said to be a prolific cause of convulsions; but this I believe is one of the chimeras in medicine. I have treated a number of cases, in which all the symptoms of worms were present, in some of which convulsions occurred, and in others they did not.

The largest number of worms I have ever known discharged from one individual, was three hundred. In this case there were no convulsions. The child was two years old; could not walk; his head enormously large, as also his thorax and abdomen, the latter tumid and pendulous; his respiration embarrassed; superior and inferior extremities in a state of marasmus.

It is evident, if worms produce convulsions by disturbing the nervous system, that the effect should have been produced in this case. In every case of convulsions I have ever seen or treated, in which worms could have been said to have acted as a remote or exciting cause, there was a strong sanguineous determination to the brain, which determination is adequate to the production of the effect, proceed from what cause it may.

Extraneous irritating substances in the stomach are often the cause of convulsions in children. I was called to a little boy during the fall of 1839, who was violently convulsed. On inquiry I found that he had been in good health up to the time of his attack. The conclusion was at once clear, that the symptoms proceeded from some irritating material in the stomach. There was violent arterial action, flushed face, and some dilatation of the pupil. I at once gave an emetic of ipecacuanha, which operated in a short time, throwing up an immense number of *percussion caps,* which were dark, and corroded by the action of the gastric juice upon them. The patient was heaving to vomit when I first saw him. So soon as the ipecacuanha had operated, I administered a full dose of oil, which purged him freely, and in twenty-four hours he was as well as ever.

In the same season I was called in great haste to see a lad four years old. On reaching the place, I found him in the most frantic, wild, convulsed condition I have ever witnessed, before or since. On examination, as in the preceding case, I found that he had been in perfect health, to all appearance, up to the period of his attack. My conviction, as in the preceding case, was that the symptoms proceeded from poison in the stomach or other offending matter. The symptoms were of a most violent character; pulse rapid, skin hot, face flushed, pupil very much dilated, wild delirium, and every voluntary muscle of the system convulsed. I immediately administered an emetic, which was with difficulty poured down him. It operated in a short time, and the ejections being retained, were found, on examination, to contain a number of the seed of the datura stamonium. After the operation of the emetic, the symptoms assumed a milder form. The emetic was repeated, with copious diluent drinks, and when it had operated effectually, I gave a full dose of oil, which operated well. Under this treatment the convulsions subsided. From the time I saw him first, until he was relieved, was eight or ten hours, and such was the violence of the determination to the brain and eyes, that the latter organs took on inflammatory action, requiring venesection, topical and general blisters to the temples, and all the remedies usual in the treatment of conjunctivitis.

*Irritation from dentition*, is also said to be a cause of spasmodic affections in children. But this is a point upon which there is some diversity of opinion among physicians. That an irritable condition of the jaws often occurs during the process of dentition is well understood, but it is rather a consequence of disorder in other parts than a cause of convulsions. The probability of this opinion is favored by the fact that disease from teething is manly confined to the summer and fall months, during which seasons children, in common with all other persons, are constantly exposed to causes calculated to derange the general health of individuals and communities. The process of teething is not confined to any particular season; it appears in all seasons, and numbers of children pass through the different stages of it without suffering inconvenience. It is therefore not a cause of convulsions, but may proceed from the same internal pathological condition which is the proximate cause of them. I have attended cases of convulsions occurring in the summer and fall seasons of the year, in which there was redness and swelling of the gums, which was said by all who saw them, to be the cause of the disease. If this had been true, my practice was certainly empirical; nevertheless, I afforded relief by the use of those means, the direct effects of which were to curb arterial action, establish secretion, and reduce plethora. In some instances where the redness and swelling of the gums were considerable, I have used the scarificator with considerable advantage by reducing the topical congestion of the part. I believe, therefore, that malaria is the efficient remote cause of all those cases of convulsions which are attributed to irritation from dentition, aided, in some instances, by the sensible properties of the atmosphere, but principally by humidity of that fluid. Many facts might be cited in support of this opinion—suffice it to say, that convulsions in children are not uncommon occurrences in intermittent fevers, particularly in the cold stage of this disease. I have known them to occur frequently, and in one case I recollect distinctly there was a regular periodical return of convulsions in place of the chill, succeeded by increased action, &c., which was relieved by the same remedies that are necessary in the treatment of intermittents.

The great indications of treatment to be observed, are, to obviate as far as possible the influence of the remote causes; to determine action from the brain to other parts of the system; to establish secretory action in every organ. To protect the brain from too powerful a determination of blood to, and congestion of its vessels, is one of the most important indications to be fulfilled in the treatment of convulsions. This indication is most promptly fulfilled, as my experience and observation induce me to believe, by the use of the tepid salt-water bath, at the same time pouring cold water on the head. The judgment of the physician is to determine the length of time necessary to keep the patient in the bath, and the number of times it should be repeated. Most generally, however, I have found five minutes to answer the purpose of quieting the symptoms, and have found it necessary to repeat the remedy three or four times during the night or day, as the case may be—sometimes using the cold application without the bath.

It is proper to remark, that frictions increase the value of the remedy, and these should be performed while the patient is in the bath; but it should always be preceded by venesection, if the pulse be full, tense, or quick. The preceding remedies are not particularly designed to establish secretions, nor are they calculated especially to act on the secretory system; yet they do in some instances promote it to a greater or less extent. But they invariably reduce the system to the state, which is the most favorable to the action of the remedies which induce secretion. I have been in the habit of giving a powder composed of one grain of calomel and one of ipecacuanha, either in pill or powder, and repeated every hour until an ordinary dose has been given. I allow the patient to rest quietly three or four hours, when, if the medicine has not operated, I aid it by injections, warm poultices to the abdomen, or an ordinary dose of oil or salts. The first class of auxiliaries I prefer, if they will answer the purpose. If the disease is conceived to proceed from cold, advantage may be derived from the proper use of diaphoretics, of which I have found the pulvis Doveri to fulfil the indications as promptly and effectually as any I have ever used. In prescribing this remedy for the cure of this disease, which is strictly inflammatory, I have found it necessary, in order to insure its diaphoretic effect, to combine with it double the quantity of ipecacuanha, and to administer it in such doses and so frequently, as to induce nausea.

The above is an imperfect outline of the treatment necessary in convulsions in children, when they proceed from cold, or malaria, and are not complicated with other diseases.

If worms be present, anthelmentics are indicated, while the convulsions are to be controlled by venesection and the warm bath.

If, after the convulsions are relieved, and the worms discharged, the patient fall into a state of languor tending to coma—the skin being cold and inactive, and the pulse weak and frequent, further treatment is called for. In such cases, I have found an ordinary dose of calomel to answer an excellent purpose. But if there be general debility and relaxation of the chylopoetic viscera, with a tendency to serous discharges, if they be not already present, more advantage is to be derived from minute doses of that article frequently repeated. I have used it much in both modes, and, when given in the latter, I have never known it fail to establish biliary secretion. Care, of course, is to be taken to prevent ptyalism where the remedy is continued for any length of time, and the only safety consists in seeing that it operates well.

My views of the nature and treatment of this interesting form of disease are submitted to the profession, not in the hope that they contain any thing new, but to contribute what I may to the establishment of a rational and sound practice.

*May 27th*, 1840.

